# Advanced glycation end products induce chemokine/cytokine production via activation of p38 pathway and inhibit proliferation and migration of bone marrow mesenchymal stem cells

**DOI:** 10.1186/1475-2840-9-66

**Published:** 2010-10-22

**Authors:** Ke Yang, Xiao Qun Wang, Yu Song He, Lin Lu, Qiu Jing Chen, Jing Liu, Wei Feng Shen

**Affiliations:** 1 Department of Cardiology, Ruijin Hospital, Jiaotong University School of Medicine, Shanghai 200025, P.R. China; 2 Institute of Cardiovascular Diseases, Jiaotong University School of Medicine, Shanghai 200025, P.R. China

## Abstract

**Background:**

Advanced glycation products (AGEs), as endogenous inflammatory mediator, compromise the physiological function of mesenchymal stem cells (MSCs). MSCs have a potential role in cell replacement therapy in acute myocardial infarction and ischemic cardiomyopathy. However, mechanisms of AGEs on MSCs are still not unveiled.

**Methods:**

Reactive oxygen species (ROS), genes regulation, cell proliferation and migration have been detected by AGE-BSA stimulated MSCs.

**Results:**

We found that *in vitro *stimulation with AGE-BSA induced generation of reactive oxygen species (ROS), and inhibited dose-dependently proliferation and migration of MSCs. Microarray and molecular biological assessment displayed an increased expression and secretion of Ccl2, Ccl3, Ccl4 and Il1b in a dose- and time-dependent manner. These chemokines/cytokines of equivalent concentration to those in conditioned medium exerted an inhibitory effect on MSC proliferation and migration after stimulation for 24 h. Transient elevation of phospho-p38 in MSCs upon AGE-BSA stimulation was blocked with p38 inhibitor.

**Conclusions:**

The study indicates that AGE-BSA induces production of chemokines/cytokines in a dose- and time-dependent manner via activation of ROS-p38 mediated pathway. These chemokines/cytokines exert an inhibitory effect on MSC growth and migration, suggesting an amplified dysfunction of MSCs by AGEs.

## Background

Emerging evidence has demonstrated that cell-based therapy including mesenchymal stem cells (MSCs) for acute myocardial infarction or ischemic cardiomyopathy holds promise [1-3]. MSCs, isolated from bone marrow, exhibit a high capacity of *ex vivo *expansion, allowing further biological modifications and clinically huge-dose preparation of the cells. Besides, MSCs are characterized by great potential to transdifferentiate into cardiomyocytes and vascular-like structure [4-6].

Diabetes is associated with adverse outcome after myocardial infarction [7]. Not unexpectedly, the effects of improving left ventricular function and reducing infarct size after stem cell therapy, which are observed in non-diabetes, have been significantly attenuated or bleached in diabetic patients with acute myocardial infarction [8]. Type 2 diabetes mellitus (T2DM) not only decreases the abundance of bone marrow derived CD133+ stem cells following acute myocardial infarction, but also limits their activation [9]. However, the abnormal profiles of MSCs in diabetes and disease-related mechanisms have been less clarified.

One of the reasons for stem cell dysfunction is due to exposure of advanced glycation end products (AGEs) in diabetic milieu. Previous studies have shown that AGEs are significantly associated with diabetic cardiovascular complications and worse prognosis [10,11]. *In vitro *stimulation with glyceraldehydes- or glycolaldehyde-modified albumin reduces proliferation of MSCs, and increases intracellular generation of reactive oxygen species (ROS) and number of apoptotic cells, with accompanying inhibition of adipogenic or chondrogenic differentiation [12]. It remains unclear if glycated protein could amplify the inflammatory response in MSCs and inhibit proliferation and migration of these cells.

The present study has shown that AGE-BSA dose-dependently inhibited proliferation and migration of MSCs via ROS-p38 MAPK-mediated pathway. Microarray analysis and molecular biological approach of gene expressions displayed increased expression and secretion of chemokines and cytokines including CC chemokine ligand (Ccl) 2, Ccl3, Ccl4 and interleukin (Il)-1 beta. Notably, these proinflammatory factors of equivalent concentration to those in conditioned medium (AGE-BSA, 200 ug/ml) functioned to inhibit proliferation and migration of MSCs.

## Materials and methods

The Animal Care Committee of the National Cardiovascular Center approved the experimental protocol.

### Cell culture

Isolation and expansion of MSCs were performed as previously described [13]. Briefly, bone marrow cells were isolated from male Sprague Dawley rats (weighing 100-150 g) by flushing out the femoral and tibial cavities with phosphate-buffered saline. Cells were grown in low glucose Dulbecco's Modified Eagle Medium, supplemented with 10% fetal bovine serum, 100 U/ml penicillin and 100 ug/ml streptomycin (Gibco, NY, USA). These cells were proved to be positive for CD29 (Biolegend, CA, USA) and CD90 (eBioscience, CA, USA) surface markers and negative for CD34 (Santa cruz, CA, USA) and CD45 (Abcam, Cambridge, UK) [14]. The STEMPRO osteogenesis and adipogenesis differentiation kits (Gibco) were used to detect the capacity of MSC differentiation.

### MTT assay

The proliferation of MSCs was tested by 3-(4, 5-dimethylthiazol-2-yl)-2, 5-diphenyltetrazolium bromide (MTT, Sigma-Aldrich, Mo, USA) [15]. MSCs (1 × 10^4^/well) were plated on a 96-well plate and stimulated by different factors at varying doses and time points. OD was measured by Microplate Reader (Bio-Rad, CA, USA) at 490 nm (n = 3).

### Measurement of intracellular ROS generation

Intracellular formation of ROS was evaluated using a fluorescent probe CM-H_2_DCFDA (Invitrogen, CA, USA) as previously described [16]. Briefly, cells were seeded in a 6-well plate (2 × 10^5 ^cells/well), and incubated with 10 uM CM-H_2_DCFDA for 60 min at 37°C. Cell nucleus was labeled by Hoechst 33342 (Sigma-Aldrich), and images were then taken (Olympus, Tokyo, Japan). The ROS fluorescence intensity was analyzed using Flow cytometry (BD, NJ, USA) (n = 3).

### Wound healing

Wound healing assay was performed as previously described [17]. Briefly, MSCs were grown to confluence in 60-mm^2 ^dishes and starved for 24 h. After completion of a linear wound, medium was changed to fresh DMEM with or without addition of factors. At 0 and 24 h, images were taken (Olympus). The area of each dish was measured using IMAGE-PRO PLUS Version 6.2 (Media Cybernetics, MD, USA).

### Boyden chamber assay

Chemotaxis was measured using a 48-well Transwell plate (Millipore, MA, USA). MSCs at 60-70% confluence were starved overnight in serum-free DMEM, trypsinized, and resuspended in serum-free DMEM (3 × 10^4 ^cells/300 ul). The cell suspension was added to the upper chamber, and the bottom chamber was filled with DMEM with 10% FBS or serum-free medium. The chamber was incubated at 37°C in a CO_2 _incubator for 5 h, then the filter was removed and nonmigrated cells were scraped from the upper surface. The migrated cells were stained and treated with lysis buffer, and number of cells was then quantified by OD 560 nm measurement.

### Microarray analysis of gene expression

MSCs (5 × 10^5^) were plated on 6-cm dishes and cultured with AGE-BSA (200 ug/ml) (Calbiochem, Darmstadt, Germany) for 12 and 24 h. Total RNA was extracted from cells using an RNeasy Mini kit (Qiagen, Hilden, Germany). Then, 10 ug of total RNA was reverse-transcribed to produce biotin-labeled cRNA, with GeneChip One-Cycle Target Labeling and Control Reagents (Affymetrix, CA, USA). After fragmentation, 10 ug of cRNA was hybridized with GeneChip Rat Genome 230 2.0 Array. GeneChips were then scanned using a GeneChip Scanner 3000. Normalization, filtering, and gene ontology analysis were performed with GeneSpring GX 7.3.1 software (Agilent Technologies, CA, USA).

The detected signals were assessed by gene hierarchical clustering of logarithmic values at each time point, and then displayed in a heatmap. Clustering was performed using Cluster 3.0 and the patterns were created and viewed using Java TreeView 1.0.13 software. Raw data from each array were analyzed using Mul-Class Dif [18-20]. Gene coexpression networks based upon normalized signal intensity were built to find interactions among genes [21]. Moreover, for assessing certain properties of the networks, k-cores in graph theory was introduced as a method of simplifying graph topology analysis [22,23].

### Quantitative real-time RT-PCR

Total RNA was extracted as described above. Briefly, 5 ug of total RNA was reverse-transcribed into cDNA using a reverse transcription system (Promega, WI, USA). PCR amplification was performed with Power SYBR Green PCR Master Mix (Applied BioSystems, CA, USA) in a StepOne (Applied BioSystems). The oligonucleotides used in quantitative real-time RT-PCR analysis are listed in Table 1. Gene expression levels were normalized with beta-actin, and data were analyzed with StepOne software v2.1 (Applied BioSystems).

**Table 1:** Primers used in real-time PCR

Gene Name	Product size (bp)	Sense primer	Anti-sense primer
β-actin	176	CGTTGACATCCGTAAAGACC	TAGAGCCACCAATCCACACA
Ccl2	280	AATGAGTCGGCTGGAGAA	GCTTGAGGTGGTTGTGGA
Ccl3	155	GCTGCCCTTGCTGTTCTT	CAAAGGCTGCTGGTCTCA
Ccl4	148	TCTCCTCCTGCTTGTGGC	GCAAAGGCTGCTGGTCTC
Il1-β	190	GGATGGTGGAGCAAGGG	GCACTGCTTCCCAGGCTT

### Measurement of chemokines/cytokines in conditioned medium

The supernatant of MSCs was collected after stimulationôand levels of Ccl2, Ccl3, Ccl4 and Il1b were measured using commercially available ELISA kits of Ccl2, Il1b(all for Invitrogen), Ccl3 (ABD Serotec, NC, USA) and Ccl4 (eBioscience).

### Effect of chemokines/cytokines on MSCs

Since the distinct effects of Ccl2, Ccl3, Ccl4 and Il1b (all for Invitrogen) on MSCs might be disturbed using conditioned medium, MSCs were therefore stimulated by commercially available chemokines/cytokines with equivalent concentration at low and high doses (as physiological or disease status [24-29]). Proliferation and migration were evaluated by MTT and wound healing assays.

### Western blot

Cells were lysed with the ProteoJET Mammalian Cell Lysis Reagent (Fermentas, MD, USA) to extract cytoplamic proteins. Equal amounts of protein extracts were subjected to 12% SDS/PAGE and blotted onto a poly (vinylidene difluoride) membrane. The membrane was blocked and probed overnight at 4°C with antibodies against total or phosphorylated p38 (Cell Signaling Technology, MA, USA), followed by incubation with horseradish peroxidase-conjugated secondary antibodies for 1 h at room temperature. Blots were developed using an ECL detection system (Millipore, MA, USA). Each image was captured and the intensity of each band was analyzed with Quantity One (Bio-Rad).

### AGE-BSA stimulation and p38 inhibition

MSCs were seeded on 96- or 6-well culture plates for MTT, microarray assay, ROS measurement, cytokine/chemokine detection, RT-PCR or western blot analysis. Cells were incubated with AGE-BSA (Calbiochem) at varying concentrations and up to different time points.

To probe the effect of P38 inhibition on activation of AGE-BSA stimulated cells, MSCs were pretreated with SB203580 (20 uM) (Merck, Darmstadt, Germany) 1 h before AGE-BSA stimulation. The concentration of inhibitor used was based upon dose-response experiments (data not shown), with the maximal inhibitory effect. For each experiment, cell viability was always more than 90%. Cell-free supernatants were collected and stored at -80°C until analysis.

### Statistical analysis

All values are expressed as mean ± SD. Student's paired t test was performed for comparison of paired samples, and ANOVA was used for multiple group comparisons, followed by Friedman's posttest. A probability (p) value < 0.05 was considered significant.

## Results

### AGE-BSA induced ROS accumulation and attenuated proliferation of MSCs

**Figure 1:**
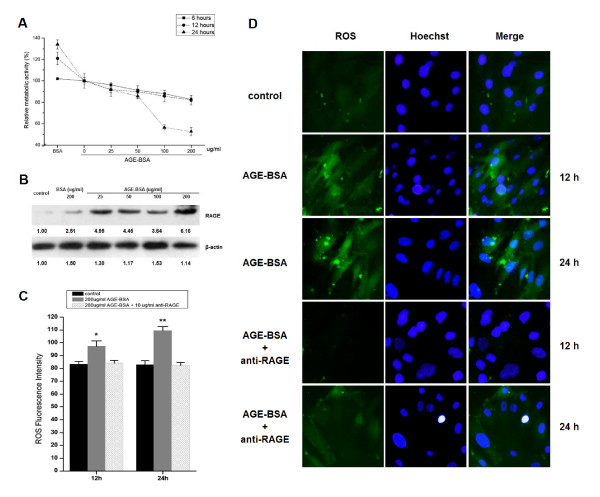
**Effects of AGE-BSA stimulation on MSC proliferation and ROS production**. **(A) **MSCs were stimulated by AGE-BSA and BSA (as negative control) for MTT testing (mean ± SD, n = 3). **(B)** AGE-BSA treated MSCs, and RAGE has been detected. **(C)** MSCs stimulated by AGE-BSA for 12 and 24 h were labeled with CM-H2DCFDA. ROS fluorescence intensity was analyzed using flow cytometry (mean ± SD, n = 3; *P < 0.05, **P < 0.01 vs. control). **(D)** MSCs were labeled with CM-H2DCFDA and Hoechst 33342. Images were taken with different fluorescence wavelengths (200×).

Fluorescence-activated cell sorting (FACS) showed that MSCs were negative for CD34 and CD45, and strongly expressed CD29 and CD90. When cultured in adipogenic or osteogenic medium, MSCs were differentiated into adipocytes or osteoblasts (see Additional File 1; Figure S1). The MTT assay showed that AGE-BSA time- and dose-dependently inhibited proliferation of MSCs, with concentration of 200 ug/ml exhibiting remarkable effects at 24 h (p < 0.05, Figure 1A). AGE-BSA regulated RAGE expression (Figure 1B). After stimulation with AGE-BSA (200 ug/ml) at 12 or 24 h, profuse ROS generation was observed in MSCs (both p < 0.05) (Figure 1C and Figure 1D).

### AGE-BSA attenuated migration of MSCs

**Figure 2:**
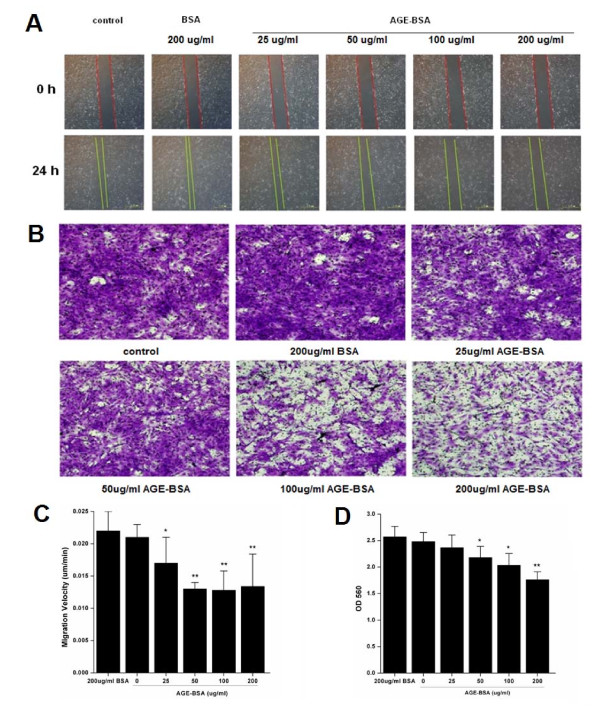
**Effects of AGE-BSA stimulation on MSC migration**. **(A) **MSCs stimulated by AGE-BSA and BSA (as negative control) for 24 h. Images were taken (40×). **(B) **Velocity of MSC migration was measured in um/min. **(C) **MSCs stimulated by AGE-BSA and BSA for 24 h were labeled with buffer of cell stain. Images were taken (200×). (D) Lyses were quantified by OD 560 nm measurement. (mean ± SD, n = 3; *P < 0.05, **P < 0.01 vs. un-stimulated cells).

Wound healing assay revealed that AGE-BSA produced a concentration-dependent decrease in migration of MSCs (Figure 2A and 2B). Similarly, AGE-BSA at these concentrations obviously inhibited transwell migration (all p < 0.05) (Figure 2C and 2D).

### Gene expression regulated by AGE-BSA

**Figure 3:**
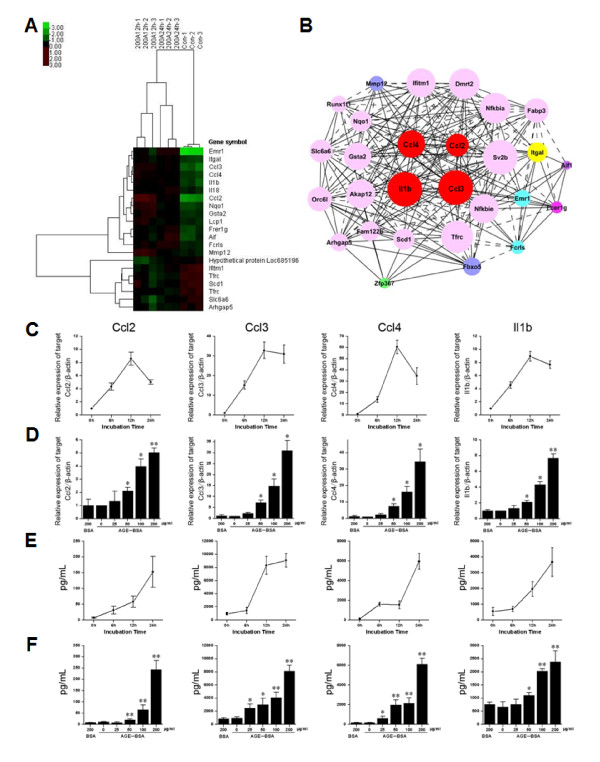
**Gene expression regulated by AGE-BSA**. **(A) **Ccl2, Ccl3, Ccl4 and Il1b showed significant difference (p < 0.01) in MSCs stimulated by AGE-BSA for 12 and 24 h using Cluster 3.0 software. **(B) **k-core of gene expression was analyzed by GeneRelNet, and net of gene co-expression was drawn. **(C) **Time- and **(D) **dose-dependent expression of chemokines/cytokines stimulated by AGE-BSA. β-actin was used to normalize the results. **(E) **Time- and **(F) **dose-dependent chemokine/cytokine production stimulated by AGE-BSA (mean ± SD, n = 3; *P < 0.05, **P < 0.01 vs. un-stimulated cells). (mean ± SD, n = 3; *P < 0.05, **P < 0.01 vs. un-stimulated cells).

The genes with significant change (p < 0.05 and FDR < 0.05) were selected (Table 2). Cluster analysis was shown in Figure 3A. Coexpression network of AGEs-induced genes was built (Figure 3B), in which Ccl2, Ccl3, Ccl4 and Il1b were located centrally.

**Table 2:** Multi-class differentiation analysis of genes

Description	Gene symbol	p-value	FDR
EGF-like module containing, mucin-like, hormone receptor-like 1	Emr1	7.00E-07	0.0003
Chemokine (C-C motif) ligand 3	Ccl3	1.00E-06	0.0003
Chemokine (C-C motif) ligand 2	Ccl2	1.04E-05	0.0022
Interleukin 1β	Il1b	2.27E-05	0.0032
Chemokine (C-C motif) ligand 4	Ccl4	2.43E-05	0.0032
Transferrin receptor	Tfrc	3.18E-05	0.0034
Integrin alpha L	Itgal	0.0003331	0.0309
NAD(P)H dehydrogenase, quinone 1	Nqo1	0.000419	0.0340
Fatty acid binding protein 3, muscle and heart	Fabp3	0.0004791	0.0341
Nuclear factor of kappa light polypeptide gene enhancer in B-cells inhibitor, alpha	Nfkbia	0.000525	0.0341
Transferrin receptor	Tfrc	0.0007492	0.0442
Nuclear factor of kappa light polypeptide gene enhancer in B-cells inhibitor, epsilon	Nfkbie	0.0010249	0.0512
Glutathione S-transferase A2	Gsta2	0.0010941	0.0512
Interferon induced transmembrane protein 1	Ifitm1	0.0011035	0.0512
Fc fragment of IgE, high affinity I, receptor for; gamma polypeptide	Fcer1g	0.0015853	0.0686
Fc receptor-like S, scavenger receptor	Fcrls	0.0027302	0.1107
Synaptic vesicle glycoprotein 2b	Sv2b	0.003675	0.1403
Doublesex and mab-3 related transcription factor 2	Dmrt2	0.0078799	0.2841
Matrix metallopeptidase 12	Mmp12	0.0091006	0.3109
Stearoyl-Coenzyme A desaturase 1	Scd1	0.0101781	0.3303
Runt-related transcription factor 1; translocated to, 1 (cyclin D-related)	Runx1t1	0.0138214	0.4254
Allograft inflammatory factor 1	Aif1	0.0144188	0.4254
Rho GTPase activating protein 5	Arhgap5	0.0162094	0.4574
F-box protein 5	Fbxo5	0.0174488	0.4718
Zinc finger protein 367	Zfp367	0.0237975	0.6178
Origin recognition complex, subunit 6 like (yeast)	Orc6l	0.0252814	0.6311
A kinase (PRKA) anchor protein 12	Akap12	0.0334329	0.7749
Family with sequence similarity 122B	Fam122b	0.0361249	0.8085
Solute carrier family 6 (neurotransmitter transporter, taurine), member 6	Slc6a6	0.0465527	0.8886

Microarray assay revealed an elevated expression of Ccl2, Ccl3, Ccl4 and Il1b after induction of AGEs. Real-time PCR verified a time- and dose-dependent increase of these chemokines/cytokines after incubation with AGE-BSA (Figure 3C and Figure 3D). As expected, the levels of Ccl2, Ccl3, Ccl4 and Il1b displayed a time- and dose- dependent increase (n = 3, p < 0.01) (Figure 3E and 3F).

### Activity of p38 pathway and chemokines/cytokines

**Figure 4:**
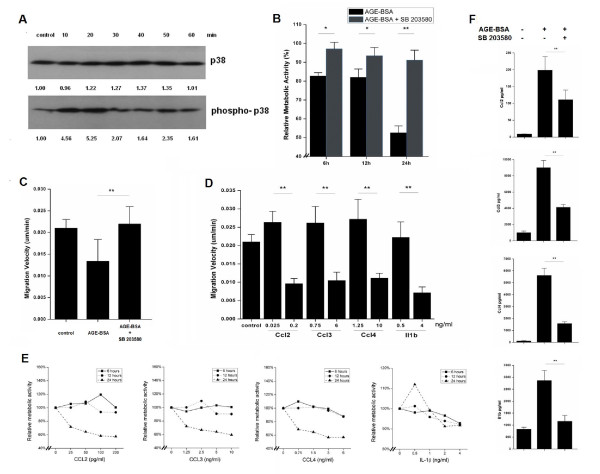
**Effects of AGE-BSA on MSCs via p38 pathway**. **(A) **MSCs were incubated with AGE-BSA and p38 phosphorylation was assessed (gray value). **(B) **Proliferation of MSCs stimulated by AGE-BSA and SB 203580 was determined by MTT. **(C) **Migration of MSCs stimulated by AGE-BSA and SB 203580 was determined by wound healing assay. **(D) **Ccl2, Ccl3, Ccl4 and Il1b secretion of MSCs stimulated by AGE-BSA and SB 203580 was evaluated. (mean ± SD, n = 3; *P < 0.05, **P < 0.01 vs. AGE-BSA stimulated cells) **(E) **MSCs stimulated with different doses and times of Ccl2, Ccl3, Ccl4 and Il1b were determined by MTT (mean ± SD, n = 3). **(F) **MSCs were stimulated by different doses of chemokines/cytokines, and migration velocity was calculated by wound healing assay (um/min). (mean ± SD, n = 3; *P < 0.05, **P < 0.01 vs. cells stimulated with low dose).

MAP kinases including p38, ERK 1/2 and JNK showed (see Additional File 2; Figure S2) that only p38 pathway affected proliferation and migration of MSCs. Stimulation with AGE-BSA (200 ug/ml) resulted in increased phosphorylation of p38 at 20, 30 and 50 min (Figure 4A). In contrast, blockage of p38 by SB203580 significantly attenuated effects of AGE-BSA on proliferation, migration and chemokine/cytokine secretion of MSCs (Figure 4B, 4C and 4D). Notably, a time- and dose-dependent inhibition of cell proliferation and migration were induced by Ccl2, Ccl3, Ccl4 and Il1b (Figure 4E and 4F)

## Discussion

The present study demonstrates that AGE-BSA inhibits proliferation and migration of MSCs, and induces production of chemokines/cytokines in a dose- and time-dependent manner via activation of ROS-p38 mediated pathway. Furthermore, these chemokines/cytokines exert an inhibitory effect on MSCs growth and migration, suggesting an amplified dysfunction of MSCs by AGEs stimulation.

Interaction of AGEs and receptor for AGEs (RAGE) relays cell surface signals to various intracellular pathways including p38, JNK/STAT and other mitogen-activated protein kinases. Activation of p38 pathway is responsible for a myriad of transcriptional programs leading to enhance expression of pro-inflammatory cytokines [24-26]. Over-production of intracellular ROS results in inflammatory activation and impairment of physiological function [27,28], and constitutes a key element in diabetic pathophysiology. In this study, we observed an increased ROS production and elevated p38 phosphorylation in MSCs by AGE-BSA stimulation, accompanying with reduced proliferation and migration and increased pro-inflammatory cytokine/chemokine production in MSCs. The extent of MSC abnormalities was closely related to the dose of AGEs, whereas blockage of p38 reverses these pathological changes. Collectively, our results suggest that AGEs cause abnormal growth and migration of MSCs, and trigger production of pro-inflammatory factors via generation of ROS and activation of p38 pathway.

Recently study demonstrated that p38 pathway activation has been associated with interleukin-6 (IL-6), cyclooxygenase-2 (COX-2) and CCL2 expression increased in the heart. The long-term activation of p38 also regulated post-infarct cardiac remodeling in type 2 diabetes. Glucagon-like peptide-1 is an incretin hormone that effects on glucose metabolism and may involve activation of p70s6 kinase, ERK1/2, and p38 MAPK. All of these research indicated that p38 involved in coronary heart disease [29-31].

In this study, a dose- and time-dependent over-expression of Ccl2, Ccl3, Ccl4 and Il1b in MSCs by AGE-BSA stimulation was evident. Previous studies have demonstrated that *in vivo *levels of these pro-inflammatory chemokines/cytokines are significantly associated with type 1 and type 2 diabetes, diabetic complications, and clinical outcomes [32-40]. Consistent with previous findings, our study further showed that chemokines/cytokines of concentration equivalent to those in conditioned medium exerted an inhibitory effect on MSC growth and migration. Notably, the concentrations of these pro-inflammatory factors in this experiment were also similar to *in vivo *levels in diabetic patients [[33,35,36] and [39]]. Thus, autocrine-released chemokines/cytokines from MSCs by AGEs stimulation and those from other sources jointly contributed to impairment of cellular function.

Based upon these observations, we hypothesized that migrated or transplanted MSCs may be functionally dysregulated in diabetic, hypoxic and inflammatory environment and exhibit low growth rate and impaired function. Inflammation is intensified with significant release of pro-inflammatory chemokines/cytokines from MSCs. These factors, with diabetic milieu, in return further damage cellular function, which mitigate the therapeutic effects of MSCs in diabetes. Interestingly, mildly elevated levels of Ccl2, Ccl3, Ccl4 and Il1b stimulated MSC homing and promote repairing of the damage tissues in non-diabetic physiological conditions [41-46].

## Conclusions

The present study demonstrates an inhibitory effect of AGEs on MSC proliferation and migration via ROS-p38-mediated pathway and production of pro-inflammatory chemokines/cytokines. Measures used for glycemic control and anti-inflammation should be emphasized especially in patients with diabetes undergoing cell therapy with MSCs.

## List of abbreviations

MSCs: mesenchymal stem cells; AGEs: advanced glycation end products; T2DM: Type 2 diabetes mellitus; ROS: reactive oxygen species; Ccl2: chemokine (C-C motif) ligand 2; Ccl3: chemokine (C-C motif) ligand 3; Ccl4: chemokine (C-C motif) ligand 4; Il1b: interleukin 1 beta; MTT: 3- (4, 5- dimethylthiazol- 2- yl)- 2, 5-diphenyltetrazolium bromide; RT-PCR: reverse transcription polymerase chain reaction; FACS: Fluorescence-activated cell sorting; FDR: False discovery rate; RAGE: advanced glycosylation end product-specific receptor; SB203580, 4-(4-fluorophenyl)-2-(4-methylsulfinylphenyl)-5-(4- pyridyl) imidazole.

## Competing interests

The authors declare that they have no competing interests.

## Authors' contributions

KY and XW carried out the molecular research, participated in cell biology experiment and drafted the manuscript. YH carried out the microarray test, participated in cell biology experiment. QC and JL carried out immunoassays, participated in molecular research. KY, XW, YH and LL participated in the design of the study and performed the statistical analysis. WS conceived of the study, and participated in its design and coordination. All authors read and approved the final manuscript.

## Supplementary Material

Additional File 1 **Fig. S1 Characterization of isolated MSCs**. **(A) **STEMPRO Osteogenesis differentiation medium induced MSC osteogenesis, stained by alkaline phosphatase. STEMPRO Adipogenesis differentiation medium induced MSC adipogenesis, stained by oil red O. The entire image was taken (10×). **(B) **Flow cytometry shows the passage 3 MSCs were negative for reactivity to antigens CD45 and CD34, and positive for reactivity to antigens CD90 and CD29. Click here for file

Additional File 2 **Fig. S2 Effect of other MAPK pathway stimulated by AGE-BSA**. **(A) **ERK1/2 and JNK phosphorylation of MSCs incubated with AGE-BSA (200 ug/ml for 0, 10, 20, 30, 40, 50 and 60 min) was determined (gray value). **(B) **Proliferation of MSCs incubated with AGE-BSA (200 ug/ml) and PD 98059 (20 uM) or JNK inhibitor Ⅱ (10 nM) for 0, 12 and 24 h was assessed by MTT. **(C) **Migration of MSCs incubated with AGE-BSA (200 ug/ml) and PD 98059 (20 uM) or JNK inhibitor Ⅱ (10 nM) for 24 h was determined by wound healing assay. (mean ± SD, n = 3; P â 0.05 vs. AGE-BSA stimulated cells). Click here for file
