# Supplementary material for: Advanced glycation end products induce chemokine/cytokine production via activation of p38 pathway and inhibit proliferation and migration of bone marrow mesenchymal stem cells
Source: Cardiovasc Diabetol. 2010 Oct 22;9:66. doi: 10.1186/1475-2840-9-66 (PMC2987998; doi:10.1186/1475-2840-9-66)
Supplement: Additional File 1 — Fig. S1 Characterization of isolated MSCs. (A) STEMPRO Osteogenesis differentiation medium induced MSC osteogenesis, stained by alkaline phosphatase. STEMPRO Adipogenesis differentiation medium induced MSC adipogenesis, stained by oil red O. The entire image was taken (10×). (B) Flow cytometry shows the passage 3 MSCs were negative for reactivity to antigens CD45 and CD34, and positive for reactivity to antigens CD90 and CD29. [file 1475-2840-9-66-S1.DOC]

**Fig. S1**
