# Supplementary figures and images for: Advanced glycation end products induce chemokine/cytokine production via activation of p38 pathway and inhibit proliferation and migration of bone marrow mesenchymal stem cells
Source: Cardiovasc Diabetol. 2010 Oct 22;9:66. doi: 10.1186/1475-2840-9-66 (PMC2987998; doi:10.1186/1475-2840-9-66)

**Fig. S2**


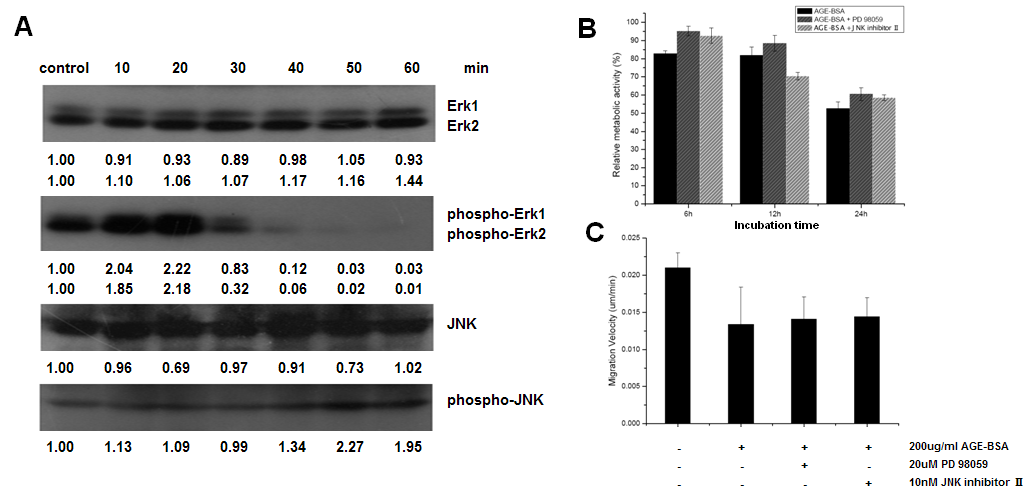

Supplement: Additional File 2 — Fig. S2 Effect of other MAPK pathway stimulated by AGE-BSA. (A) ERK1/2 and JNK phosphorylation of MSCs incubated with AGE-BSA (200 ug/ml for 0, 10, 20, 30, 40, 50 and 60 min) was determined (gray value). (B) Proliferation of MSCs incubated with AGE-BSA (200 ug/ml) and PD 98059 (20 uM) or JNK inhibitor Ⅱ (10 nM) for 0, 12 and 24 h was assessed by MTT. (C) Migration of MSCs incubated with AGE-BSA (200 ug/ml) and PD 98059 (20 uM) or JNK inhibitor Ⅱ (10 nM) for 24 h was determined by wound healing assay. (mean ± SD, n = 3; P â 0.05 vs. AGE-BSA stimulated cells). [file 1475-2840-9-66-S2.DOC]
